# The Clinicopathologic and Prognostic Significance of c-Myc Expression in Hepatocellular Carcinoma: A Meta-Analysis

**DOI:** 10.3389/fbinf.2021.706835

**Published:** 2021-10-12

**Authors:** Zhao Min, Zhang Xunlei, Chen Haizhen, Zhao Wenjing, Yu Haiyan, Lu Xiaoyun, Zhou Jianyun, Chen Xudong, Shen Aiguo

**Affiliations:** ^1^ Department of Pathology, Affiliated Tumor Hospital of Nantong University, Nantong, China; ^2^ Department of Oncology, Affiliated Tumor Hospital of Nantong University, Nantong, China; ^3^ Cancer Research Center, Affiliated Tumor Hospital of Nantong University, Nantong, China

**Keywords:** HCC, c-Myc, clinical pathology, prognostic, meta-analysis

## Abstract

**Background:** The incidence and mortality rates of hepatocellular carcinoma (HCC) are increasing worldwide. Therefore, there is an urgent need to elucidate the molecular drivers of HCC for potential early diagnosis and individualized treatment. Whether c-Myc expression plays a role in the clinicopathology and prognosis of patients with HCC remains controversial. This meta-analysis aimed to survey the prognostic role of c-Myc in HCC.

**Methods:** We searched PubMed, Cochrane Library, Embase, Web of Science, and Google Scholar databases for studies published through March 2020 that examined the association between c-Myc expression and clinicopathology or prognosis in HCC patients. The pooled hazard ratios (HRs) and 95% confidence intervals (CIs) were used to investigate the prognostic significance of c-Myc expression. Odds ratios were calculated to evaluate the association between c-Myc expression and clinicopathologic features. We also tested for publication bias.

**Results:** Our meta-analysis included nine studies with 981 patients with HCC published between 1999 and 2016. A meta-analysis of these studies demonstrated that high c-Myc expression indicated a poor overall survival (OS) (HR = 2.260, 95% CI: 1.660–3.080, and *p* < 0.001) and disease-free survival (DFS) (HR = 1.770, 95% CI: 1.430–2.450, and *p* < 0.001) in patients with HCC. However, high c-Myc expression was not associated with HBsAg, pathological type, TNM stage, or cirrhosis. We did not find any significant publication bias among the included studies, indicating that our estimates were robust and reliable.

**Conclusion:** c-Myc overexpression could predict poor OS and DFS in HCC patients. c-Myc could be a useful prognostic biomarker and therapeutic target for HCC.

## Introduction

Primary liver cancer (PLC) is a high-risk malignant tumor worldwide, with approximately one million new patients every year (Kulik and El-Serag, 2019). Hepatocellular carcinoma (HCC) is the main type of PLC, accounting for approximately 75–85% of cases (Bray et al., 2018). The incidence and mortality rates of HCC are increasing worldwide. Furthermore, most patients are diagnosed with middle- or advanced stage disease. Currently, there are no effective preventative or systemic treatments for HCC. Therefore, there is an urgent need to elucidate the molecular drivers of the initiation and progression of HCC for potential early diagnosis and individualized treatment.

c-Myc, a predominant member of the MYC family, is the most frequently activated oncogene. c-Myc is a transcription factor that binds to the enhancer box sequence CACGTG (Caforio et al., 2018) and directly and indirectly regulates more than 15% of human genes involved in multiple biological processes, including cell proliferation, differentiation, tumorigenesis, apoptosis, and metabolism (Dang, 1999; Nesbit et al., 1999; Dang et al., 2006). c-Myc gene amplification and overexpression are the main types of dysregulation in cancer. A variety of studies have reported an association between c-Myc dysregulation and the development of many malignancies, including HCC (Rapp et al., 2009; Ren et al., 2013; Fan et al., 2016). c-Myc is involved in the development of HCC, and almost 30% of human HCC samples show c-Myc gene amplification, which is correlated with protein expression; HBV, HCV, and aflatoxin, the main risk factors of HCC, have also been reported to induce overexpression of c-Myc (Xu et al., 2019). However, the ability of c-Myc overexpression to predict patient prognosis remains controversial (Ji et al., 2018). In this study, we performed a systematic review to clarify the conflicting data regarding the association between c-Myc dysregulation and HCC prognosis.

## Methods

### Literature Search Strategy

We comprehensively searched PubMed, Cochrane Library, Embase, Web of Science, and Google Scholar databases for studies published through March 2020. The following keywords were variably combined and used as the search criteria: hepatocellular cancer, HCC, liver cancer, c-Myc, and prognosis. Additional relevant references cited in the retrieved articles were also included in the evaluation.

### Eligibility Criteria

The following criteria were used to identify articles eligible for inclusion in this systematic review: 1) published in English with the full text available; 2) all patients were diagnosed with HCC based on pathology; 3) c-Myc expression levels were detected in tumor tissues by fluorescence *in situ* hybridization, genome quantitative polymerase chain reaction, immunohistochemistry, or other techniques; and 4) the clinical characteristics of c-Myc dysregulation were compared with detailed survival information, including overall survival (OS) and disease-free survival (DFS).

### Data Extraction

The available data were independently extracted by two reviewers (Zhao M and Zhang XL). Any dispute between the two reviewers was resolved by consensus or by a third reviewer (Chen HZ). The following details of each publication were extracted: the name of the first author; year of publication; country; ethnicity of the patients; number of patients; assessment method of c-Myc; cutoff value; follow-up time; clinicopathologic parameters, including sex, age, tumor size, tumor number, HBsAg, pathological type, cirrhosis, and TNM stage; OS or DFS; and outcomes of univariate and/or multivariate analyses [including hazard ratios (HRs), *p*-values, and 95% confidence intervals (CIs)]. Multivariate analysis results were selected when both univariate and multivariate analysis results were available in the same article.

### Quality Assessment

Two investigators independently conducted quality assessments for all included studies (Zhao WY and Yu HY). The Newcastle–Ottawa Quality Assessment Scale, recommended by the Cochrane Collaboration as a risk-of-bias assessment tool for observational studies, was applied to assess the quality of the included studies (Stang, 2010; Shuster, 2011). Three parameters were judged for study quality: selection, comparability, and outcome assessment. Each study was scored from 0 to 9 according to these parameters. Any disputes were resolved through discussion.

### Statistical Analysis

We used Cochrane’s Q test and *p*-values to assess the heterogeneity among the eligible studies. A chi-squared *p* value < 0.1 or an *I* ^*2*^ value > 50% was defined as statistically significant heterogeneity (Cochran, 1950; Higgins et al., 2003). When heterogeneity existed [*I* (Bray et al., 2018) ≥ 50%], a random-effects model was used; otherwise, a fixed-effects model was used. We used HRs with 95% CIs to measure the prognostic value of c-Myc in survival in HCC patients. An HR > 1 implied poorer survival for the higher c-Myc expression group. In contrast, an HR < 1 implied poorer survival for the lower c-Myc expression group. Odds ratios (ORs) and their associated 95% CIs were used to assess the relationship between c-Myc overexpression and clinicopathologic features. An OR > 1 represented poorer prognosis for the higher c-Myc expression group, whereas an OR < 1 indicated poorer prognosis for the lower c-Myc expression group. We extracted relevant data from the identified studies, and the ORs and HRs were estimated using a meta-analysis. All data analyses were performed using Revman 5.3 (RevMan, the Cochrane Collaboration) and Stata 11.0 (Stata, College Station). Sensitivity analysis was conducted to evaluate the stability of our estimates. Potential publication bias was evaluated using Begg’s funnel plot and Egger’s test (Egger et al., 1997). The *p*-values in the analysis were two-sided, and values less than 0.05 were regarded as statistically significant.

## Results

### Search Results

**FIGURE 1:**
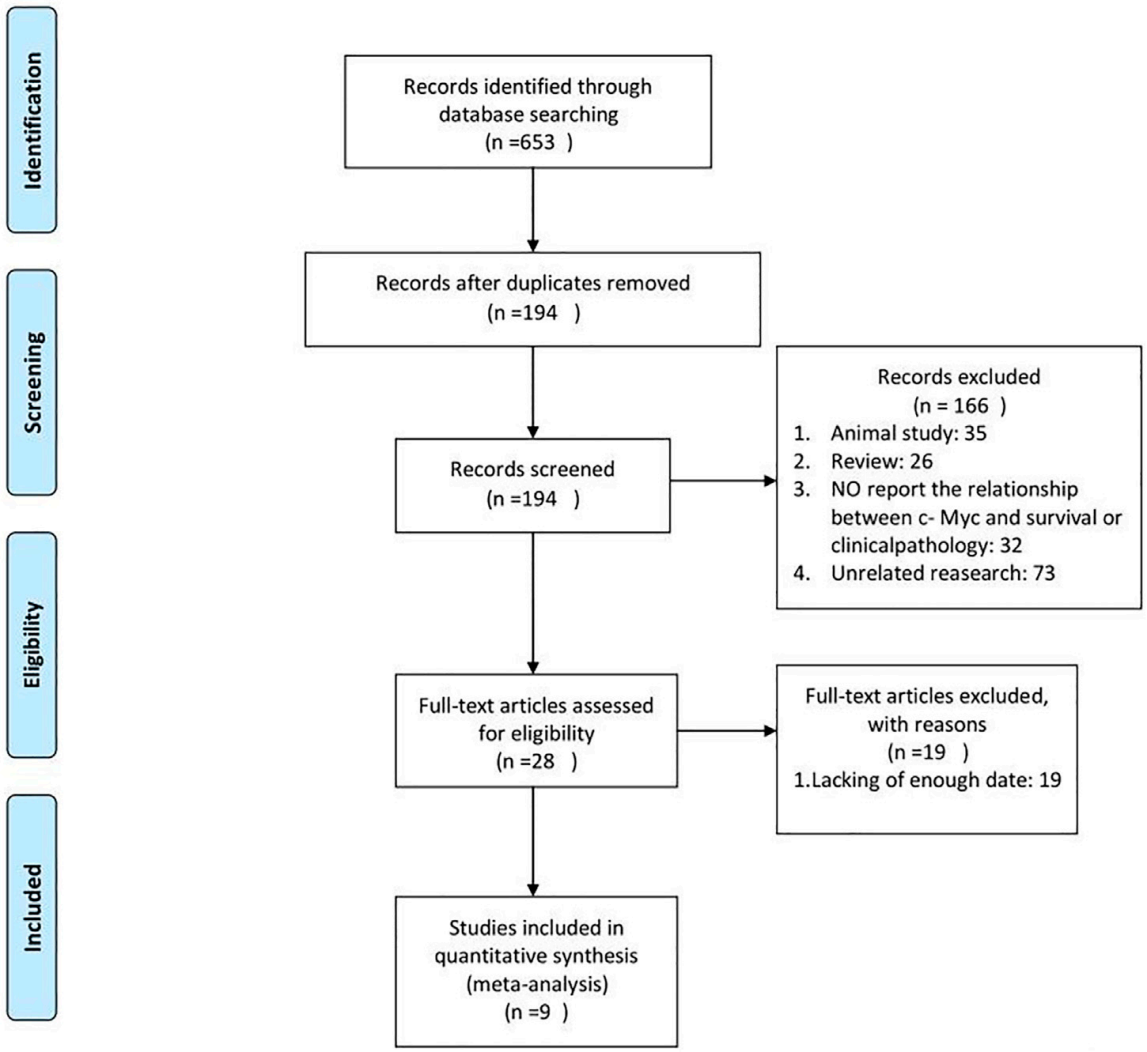
Flowchart of the literature search and study selection.

In this study, 653 articles were identified using the initial search strategy. Of these, 459 were removed because of duplication. After checking the titles and abstracts of the remaining articles, 28 full texts were further reviewed, and 9 studies were consistent with our purpose and included in the final analysis (Saegusa et al., 1993; Kawate et al., 1999; Liu et al., 2004; Jang et al., 2012; Wang et al., 2014; Huang et al., 2015; Abdou et al., 2016; Liu et al., 2016; Zheng et al., 2016). A flow diagram of the process is shown in Figure 1.

### Characteristics of the Included Studies

The characteristics of the included studies are summarized in Table 1. The 9 studies were all retrospective cohort studies published between 1999 and 2016 and included 981 patients, whose data were used in the comprehensive analysis. Seven studies (77.8%) included only Asian individuals, and two (22.2%) included both Asian and American individuals. Among the nine studies, five reported a correlation between c-Myc expression and OS or DFS, and the remaining four showed a correlation between c-Myc expression and clinicopathologic features.

**TABLE 1:** Characteristics of included studies.

Ethnicity	Treatment	Follow-up (month)	Study period	Patients (n)	Survival analysis	NOS score
Asian	Mixed	NA	2007–2015	37	OS/DFS	8
Asian	Surgery	NA	2005–2007	88	OS/DFS	8
Asian	Surgery	NA	1983–1992	40	NA	6
African	Surgery	NA	2007–2013	103	NA	6
Asian	Surgery	NA	1991–1995	42	DFS	7
Asian	Surgery	60	2007–2009	122	OS/DFS	8
American	Surgery	432	1998–2009	154	OS/DFS	8
Asian	Surgery	NA	NA	57	NA	6
Asian	Surgery	NA	NA	54	NA	6

### c-Myc Expression and Cancer Prognosis

**FIGURE 2:**
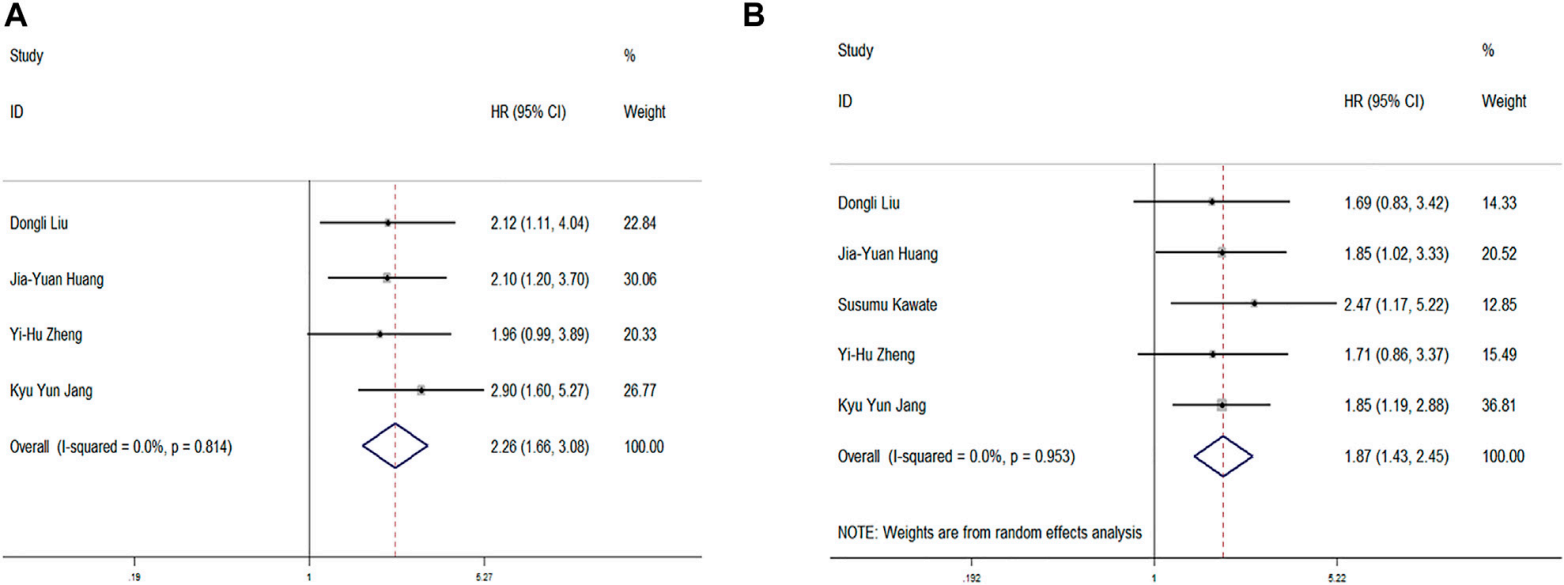
Forrest plot of HR for OS **(A)** and DFS **(B)**. Size of the square indicates the relative contribution of each study. The solid horizontal line represents 95% confidence interval of each study. The diamond indicates pooled studies.

Four studies provided data on OS. High c-Myc expression was significantly associated with poor OS (HR = 2.260, 95% CI: 1.660–3.080, and *p* < 0.001) (Figure 1). No statistically significant heterogeneity was observed (*p* = 0.814, *I* ^*2*^ = 82.1%). High c-Myc expression was also associated with poorer DFS in the random model (HR = 1.770, 95% CI: 1.430–2.450, and *p* < 0.001). However, high heterogeneity among the studies was observed (*p* < 0.001 and *I* ^*2*^ = 95.30%) (Figure 2B).

### c-Myc Expression and Clinical Features

We next calculated the association between elevated c-Myc expression and clinical parameters (Table 2). Overexpression of c-Myc was not significantly associated with positive HBsAg (OR = 0.600, 95% CI: 0.200–1.795, and *p* = 0.361), pathological type (OR = 0.867, 95% CI: 0.649–1.156, and *p* = 0.331), TNM stage (OR = 1.289, 95% CI: 0.938–1.771, and *p* = 0.117), or cirrhosis (OR = 1.160, 95% CI: 0.895–1.504, *p* = 0.261).

**TABLE 2:** Relationship between high c-Myc and the clinicopathologic features.

Variable	No. of studies	No. of patients	Effects model	OR (95%CI)	P	Heterogeneity	
						I^2^ (%)	P_H_
BbsAg (positive vs. negative)	3	383	Random	0.600 (0.200–1.795)	0.361	94.9	<0.001
Pathological type (well + mode vs. poor)	2	97	Fixed	0.867 (0.649–1.156)	0.331	0	1.424
TNM(I vs. II-III)	2	96	Random	1.289 (0.938–1.771)	0.117	57	0.127
Cirrhosis (no vs. yes)	3	197	Fixed	1.160 (0.895–1.504)	0.261	40.70	0.185

### Publication Bias and Sensitivity Analyses

**FIGURE 3:**
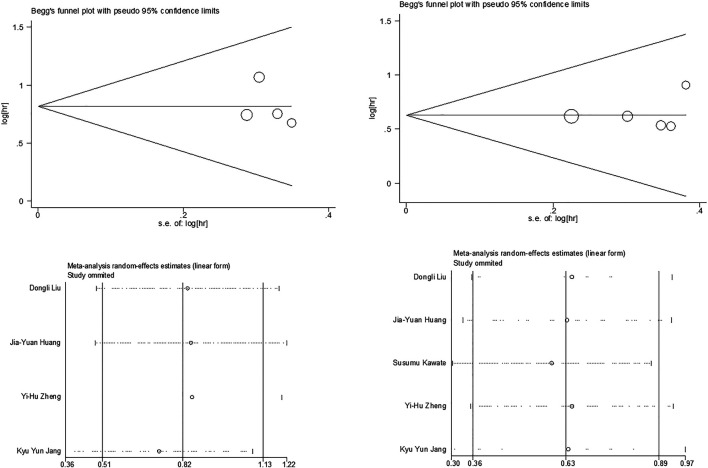
Begg’s funnel plot for publication bias tests in **(A)** OS and **(B)** DFS. Sensitivity analysis in **(C)** OS and **(D)** DFS.

We used Begg’s funnel plot and Egger’s test to examine potential publication bias. Sensitivity analysis was executed by sequentially omitting each trial one at a time. As shown in Figure 3, there was no significant publication bias for OS and DFS analysis (Egger’s test: *p* = 0.635 for OS and *p* = 0.706 for DFS). The results of the sensitivity analysis demonstrated that our assessment was accurate and reliable. Due to the limited number of articles included, sensitivity analyses of the association between c-Myc expression and clinical features were not performed.

## Discussion

This meta-analysis analyzed the association between c-Myc expression and prognosis among patients with HCC. We showed that high c-Myc expression was significantly associated with prognosis.

Cell survival and growth are promoted by c-Myc *via* the upregulation of downstream proteins involved in protein translation, cell cycle regulation, apoptosis, and metabolism (Chanvorachote et al., 2020). Cancer cell behaviors and activities, such as cell growth, survival, resistance to chemotherapies, immune surveillance, and metastasis, are also controlled by these effectors of c-Myc. c-Myc can regulate carcinogenesis and progression in many cancers, including lung cancer (Xiong et al., 2020), breast cancer (Chen et al., 2020), and colorectal cancer (Pan et al., 2020). c-Myc also plays an important role in the occurrence and development of HCC (Cheng et al., 2015; Attallah et al., 2017).

c-Myc is considered an important therapeutic target because of its dysregulation in approximately 50% of human cancers (Chen et al., 2018). Abnormal overexpression of c-Myc is related to poor prognosis in several cancers (Gustafson and Weiss, 2010), and c-Myc is overexpressed in approximately 70% of tumors. In malignant tumors, increased expression of c-Myc is associated with a malignant phenotype. Recent studies have shown that c-Myc is overexpressed in 50–100% of breast cancers (Locker et al., 1989) and 50–75% of NSCLCs (Xu et al., 2011; Jiang et al., 2016). However, the prognostic value of c-Myc in patients with HCC remains controversial. One report indicated that low expression of c-Myc could predict poor outcomes in patients with HCC after resection (Ji et al., 2018). However, another study showed that overexpression of c-Myc is correlated with poor prognosis in HCC patients (Locker et al., 1989). The Human Protein Atlas indicates that MYC is not prognostic in liver cancer. The relationship between c-Myc expression and the prognosis of HCC patients remains controversial. Therefore, it is necessary to further identify the prognostic values of c-Myc expression in HCC. We performed this meta-analysis to clarify the association of c-Myc overexpression with survival and clinicopathologic features of HCC patients.

In this meta-analysis, we included nine articles including 981 patients with HCC to accurately assess this issue. Our results indicated that overexpression of c-Myc significantly predicted poor OS and DFS in patients with HCC. This implies that c-Myc may be a biological marker of poor prognosis, and its expression can be used as a risk assessment tool for HCC patients. However, c-Myc expression was not associated with any clinicopathologic parameters we assessed, including HBsAg, pathological type, TNM stage, and cirrhosis. This may be because there were not enough patients included in our analysis. Additionally, a high degree of heterogeneity complicated the analysis. Our systematic review of a large number of researched studies shows that MYC has a known effect on the prognosis of liver cancer, although the Human Protein Atlas indicates that MYC is not prognostic in liver cancer. The reason may be that most of these studies included Asians, because of ethnic differences. Our research results indicated that c-Myc overexpression could predict poor OS and DFS in HCC. It is necessary to study the relationship between c-Myc expression and clinicopathologic characteristics in the future.

Although significant efforts have been made to conduct a comprehensive analysis, this meta-analysis has some limitations. First, all the studies in this meta-analysis were written in English. This might have caused publication bias to some extent, although we did not identify any publication bias. In the process of searching for references, we ruled out one animal study that was published in a language other than English. Therefore, there were also other reasons for exclusion. Second, our results might not apply to patients of other ethnicities because most of the patients in this study were from Asia. Third, there were not enough patients to conduct a good pooled analysis. Because of the lack of relevant data, we did not analyze all clinicopathologic features in this meta-analysis. Although our meta-analysis suggests that c-Myc is associated with HCC prognosis because of these limitations, detailed clinical studies are warranted to establish the prognostic prediction value of c-Myc in HCC in the future.

## Conclusion

This meta-analysis revealed that c-Myc overexpression predicts poor OS and DFS in patients with HCC. Our results suggest that c-Myc is a useful prognostic biomarker and therapeutic target for HCC.
